# Sub-national stratification of malaria risk in mainland Tanzania: a simplified assembly of survey and routine data

**DOI:** 10.1186/s12936-020-03250-4

**Published:** 2020-05-08

**Authors:** Sumaiyya G. Thawer, Frank Chacky, Manuela Runge, Erik Reaves, Renata Mandike, Samwel Lazaro, Sigsbert Mkude, Susan F. Rumisha, Claud Kumalija, Christian Lengeler, Ally Mohamed, Emilie Pothin, Robert W. Snow, Fabrizio Molteni

**Affiliations:** 1 Swiss Tropical and Public Health Institute, Basel, Switzerland; 2 University of Basel, Basel, Switzerland; 3 Ministry of Health, Community Development, Gender, Elderly, and Children, Dodoma, Tanzania; 4 National Malaria Control Programme, Dodoma, Tanzania; 5 Malaria Branch, Division of Parasitic Diseases and Malaria, Centers for Disease Control and Prevention, and US President’s Malaria Initiative, Dar es Salaam, United Republic of Tanzania; 6 National Institute for Medical Research, Dar es Salaam, Tanzania; 7 Clinton Health Access Initiative, New York, USA; 8 KEMRI-Welcome Trust Research Programme, Nairobi, Kenya; 9 Centre for Tropical Medicine and Global Health, Nuffield Department of Clinical Medicine, University of Oxford, Oxford, UK

**Keywords:** Malaria, Epidemiological stratification, Routine data, School surveys, Tanzania

## Abstract

**Background:**

Recent malaria control efforts in mainland Tanzania have led to progressive changes in the prevalence of malaria infection in children, from 18.1% (2008) to 7.3% (2017). As the landscape of malaria transmission changes, a sub-national stratification becomes crucial for optimized cost-effective implementation of interventions. This paper describes the processes, data and outputs of the approach used to produce a simplified, pragmatic malaria risk stratification of 184 councils in mainland Tanzania.

**Methods:**

Assemblies of annual parasite incidence and fever test positivity rate for the period 2016–2017 as well as confirmed malaria incidence and malaria positivity in pregnant women for the period 2015–2017 were obtained from routine district health information software. In addition, parasite prevalence in school children (*Pf*PR_5to16_) were obtained from the two latest biennial council representative school malaria parasitaemia surveys, 2014–2015 and 2017. The *Pf*PR_5to16_ served as a guide to set appropriate cut-offs for the other indicators. For each indicator, the maximum value from the past 3 years was used to allocate councils to one of four risk groups: very low (< 1%*Pf*PR_5to16_), low (1− < 5%*Pf*PR_5to16_), moderate (5− < 30%*Pf*PR_5to16_) and high (≥ 30%*Pf*PR_5to16_). Scores were assigned to each risk group per indicator per council and the total score was used to determine the overall risk strata of all councils.

**Results:**

Out of 184 councils, 28 were in the very low stratum (12% of the population), 34 in the low stratum (28% of population), 49 in the moderate stratum (23% of population) and 73 in the high stratum (37% of population). Geographically, most of the councils in the low and very low strata were situated in the central corridor running from the north-east to south-west parts of the country, whilst the areas in the moderate to high strata were situated in the north-west and south-east regions.

**Conclusion:**

A stratification approach based on multiple routine and survey malaria information was developed. This pragmatic approach can be rapidly reproduced without the use of sophisticated statistical methods, hence, lies within the scope of national malaria programmes across Africa.

## Background

Since 2000, there has been an unprecedented increase in funding to support the coverage of malaria interventions across Africa [1]. This renewed commitment translated into a reduction in the prevalence of malaria infection and disease burden in many parts of Africa [1, 2]. However, in recent years, progress has stalled [1, 2]. Ten countries in Africa currently account for 66% of the global malaria disease burden [3], despite increases in the distribution of effective vector control and disease management strategies. Further increases in international donor assistance are unlikely and a new model of improving investment efficiencies is required to maximize the benefits of interventions in areas likely to achieve the largest disease burden reductions. The World Health Organization (WHO) Global Technical Strategy (GTS) for malaria 2016–2030 revisited an old paradigm of stratifying sub-national malaria burden based on the analysis of past and contemporary malaria data, risk factors and the environment [4]. A major pillar of the GTS 2016–2030 is the use of accurate and timely routine data for tracking the changes in malaria epidemiology.

Since the launch of the WHO “T3” (Test, Treat, Track) initiative in 2012 [5], many African countries have increased testing rates at health facilities and are now able to provide data on malaria parasitological diagnosis performed through microscopy or malaria rapid diagnostic testing (RDT) [6]. Furthermore, countries have initiated efforts to improve their Health Management Information System (HMIS) system using the open source web-based software known as the District Health Information Software (DHIS2). Adoption of this software in many countries has facilitated the availability and access to routine malaria parasitological diagnosis data generated from health facilities which has strengthened the utilization of such data for malaria risk mapping and evaluations of intervention programmes.

Since the 1960s, the epidemiology of malaria in mainland Tanzania has been mainly described through the length of the malaria transmission seasons, urbanization, altitude and community-based parasite prevalence [7–9]. All have highlighted the extreme diversity in the potential, and empirically defined malaria transmission intensity, within the country’s borders. A more recent assembly of 10 years of community- and school-survey parasite prevalence data was used within a model-based geospatial framework to empirically highlight the heterogeneous nature of sub-national malaria transmission intensity [10–12], and used to describe the country’s epidemiological profile in the 2015–2020 National Malaria Strategic Plan (NMSP) [7]. However, these statistical models of opportunistic research data, or under-powered national household sample health surveys, provide only one means to define variations in malaria prevalence. To-date, other data, notably those generated from routine health information systems, have been underutilized and the use of epidemiological evidence to tailor sub-national malaria intervention strategies has been limited. These approaches should be data-driven, using all available routine and survey information and the stratification should be country-led [3, 13].

Since the launch of the Roll Back Malaria (RBM) initiative in 1998, the National Malaria Control Programme (NMCP) of mainland Tanzania has developed 3, 5-year NMSPs [7–9]. The third NMSP covered the period 2015–2020 [7] and aimed to reduce the national malaria prevalence from 10% in 2012 to 5% in 2017 and further to less than 1% by 2020. The initial ambition of the strategy was to sustain progress and achievements through a universal coverage of existing interventions; and during the second phase (2018 to 2020), to consolidate these achievements and explore the feasibility of a malaria pre-elimination in defined areas of the country [7].

Although progress was made towards reducing national parasite prevalence from 18% in 2008 [14] to 7% in 2017 [15], a mid-term review (MTR) in 2017 [16] recognized that a more strategic allocation of limited resources was needed to ensure continued progress in the future. The MTR was followed by a consultative meeting with global and national malaria experts [17, 18]. Recommendations from this forum together in concert with the GTS 2016–2020 [4], reiterated the need to consider tailoring intervention approaches to the sub-national local context, based on epidemiological stratification. To establish epidemiological strata at operational units of programme delivery (councils), a data-driven approach was required, that maximizes the use of survey and routine data. This paper provides an outline of the methods used to assemble infection prevalence and other malaria indicators from routine data to develop a sub-national epidemiological stratification for mainland Tanzania’s 184 councils. This paper presents the first documentation of a national effort to combine multiple epidemiological indicators from different data sources to form a composite risk stratification. The process of policy development [19] and the allocation of interventions [18] following development of this malaria risk stratification are presented elsewhere.

## Methods

### Administrative boundaries and populations at risk

In 2016, mainland Tanzania revised the administrative boundaries to 26 regions and 184 councils [20] (see Additional file 1: Figure S1). The councils represent the administrative level for operationalization and management of disease prevention and control activities and serve as resource allocation units for central government support. Councils are categorized according to population settings; 137 are rural and 47 are urban councils consisting of three types of urban authorities; city, municipal and town councils [20, 21].

The population at risk was obtained from the publicly available 2012 population and housing census in Tanzania conducted by the national bureau of statistics. Information on the population is provided by ward, the most granular level (5th administrative level), and by age and gender [22]. Population data from census conducted in 2002 and 2012 were reconstructed to the 184 councils [20] and projected for the period 2015–2017 using council annual growth rates computed from the average annual continuous growth rate formula.

### Data assembly and description of data sources

#### Survey data: school malaria parasitaemia surveys (SMPS)

In mainland Tanzania, nationwide SMPS, targeting public primary school children aged 5–16 years, were conducted in 2014–2015 and 2017. During this period, estimates of infection prevalence were available from a total of 711 sampled schools and 115,992 children (see Additional file 1: Figure S2). The survey includes malaria rapid diagnostic testing (RDT) and provides information on parasite prevalence representative at the council level [11].

#### Routine data: health facility data from HMIS/DHIS2

In 2009, the Ministry of Health, Community Development, Gender, Elderly and Children (MoHCDGEC) piloted a monitoring and evaluation (M&E) strengthening initiative to improve the HMIS, migrating from paper-based system to using the electronic DHIS2 system. DHIS2 is an open source web-based software platform for reporting, analyzing, and dissemination of data for health programmes which can be accessed by officials at all levels of health care delivery including health facility, council, regional, and national levels through registered credentials. Each month, health facilities provide monthly summary reports with data that are entered into DHIS2. Since its inception in 2013, the reporting rates from operational health facilities (Additional file 1: Figure S3) have improved dramatically with current reporting rates from Out-Patient Department (OPD) over 90%.

A focal member from the NMCP continuously engages with the M&E technical working group of the MoHCDGC to expand efforts in improving data quality through quality assurance supervisions. Additionally, the NMCP in consultation with the University of Dar es Salaam have developed an electronic platform of all available malaria data within DHIS2: the NMCP interactive malaria dashboard. The dashboard facilitates the visualization, interpretation and use of all malaria related information in the DHIS2 platform and the production of quarterly malaria bulletins for dissemination at regional, council and health facility levels.

Based on the recommendations from WHO [23], as well as consideration of the availability, frequency and robustness of malaria data, the following four routinely collected malaria indicators were selected to conduct the stratification: (1) fever test positivity rate (TPR), (2) annual parasite incidence (API), (3) confirmed malaria incidence and (4) malaria positivity rate in pregnant women.

#### Fever test positivity rate (TPR)

Monthly laboratory testing reporting tools were introduced in health facilities in October 2015 to capture the number of malaria tests performed. The RDTs were introduced in mainland Tanzania in 2009 in several rolled-out phases before country wide scale up was achieved in 2013. Currently, RDTs are the most common diagnostic tool with only a small proportion of facilities, mainly private facilities, that still use microscopy to detect malaria infections. Fever TPR was defined as the proportion of the total number of positive malaria tests among all malaria tests performed in all age groups by Pf-Pan RDT and reported by health facility laboratories. The denominator was obtained by summing the number of test positive and test negative results across all age groups. For stratification, data for the period 2016 to 2017 were used.

#### Annual parasite incidence from laboratory (API)

API is one of the core indicators recommended by WHO to be used for malaria risk stratification [24]. API presents the advantage of being easily available from the routine systems in an inexpensive manner. The API was defined as the total number of all positive malaria tests, among all malaria tests performed across all age groups by Pf-Pan RDT or microscopy at health facility laboratories per 1000 projected population per council in 2016 and 2017.

#### Confirmed malaria incidence from OPD

Ideally, the case incidence per 1000 population from OPD registers should correspond to the API calculated from the laboratory register. However, since the laboratory reporting tools (Monthly summary reports of the laboratory register) were only introduced in October 2015, overall laboratory reporting rates of health facilities in 2016 was only 49.6%. Therefore, this indicator was also considered in order to account for the low reporting rates of the monthly laboratory reports in 2016. Confirmed malaria incidence was calculated using data obtained from the OPD registers via the DHIS2 system. This included all cases diagnosed as malaria using Pf-Pan RDT or microscopy. The incidence from OPD was defined as the total number of confirmed malaria cases across all age groups per 1000 projected council population per year for the period 2015, 2016 and 2017.

#### Test positivity rate from antenatal care clinics (ANC)

Malaria testing by RDT among pregnant women attending their first visit at ANC clinics was implemented in mainland Tanzania in mid-2013 and integrated into the routine HMIS [25–27]. Tanzania is the only country in Africa to have implemented routine ANC malaria testing for surveillance. ANC TPR was defined as the proportion of the total number of positive malaria tests among all malaria tests performed by pf-pan RDT for women attending their first ANC visit. Data used for the stratification process were obtained for the complete years 2015, 2016 and 2017.

### Data processing and cleaning

Data from the SMPS required no further processing since the average prevalence per council was used. For all indicators from the health facilities, data were downloaded from DHIS2. In this analysis, the completeness for reporting was defined as the number of facility monthly reports received out of the expected number of facility monthly reports. The operational status of the health facilities during the observation period was assumed to remain constant. All reports from health facilities that were duplicated and facilities with no testing performed in all reporting months were excluded from the analysis. As the DHIS2 database is unable to distinguish zeros from missing values since it marks them as blank, it was assumed that missing values of otherwise complete reports were true zeros. Therefore, when the reporting variable indicated successful form submission, missing values of numerical variables were replaced with zero. The data utilized for stratification covered different years of completeness and coverage as summarized in Table 1.

**Table 1:** Indicators used for malaria risk stratification

Source	Indicator	Numerator	Denominator	Period^a^	Age
SMPS	Parasite prevalence	No. positive pf-pan RDT	No. Pf-Pan RDT tests performed in school children	2015, 2017	5–16 years
HMIS/DHIS2	Laboratory				
	Fever test positivity rate	No. positive pf-pan RDT	No. Pf-Pan RDT tests performed	2016–2017	All ages
	Annual parasite incidence	No. positive pf-pan RDT and microscopy	Per 1000 population^b^		
	Outpatient Department				
	Confirmed malaria incidence	No. positive pf-pan RDT, and microscopy	Per 1000 population^b^	2015–2017	All ages
	Antenatal clinic				
	Test positivity rate	No. positive pf-pan RDT	No. Pf-Pan RDT tests performed in pregnant women at first visit	2015–2017	Reproductive age

*HMIS* Health Management Information System, *DHIS2* District Health Information System 2, *RDT* malaria Rapid Diagnostic Test, *pf Plasmodium falciparum*, *SMPS* School Malaria Parasitaemia Survey

^a^January 1st to December 31st of the corresponding year; ^b^Based on population estimates from the 2012 census

Microsoft Excel was used for cleaning and analysis of the data downloaded from DHIS2 as well as for conducting the stratification. Stratified maps were produced using QGIS software version 3.0.3 [28].

### Stratification

The stratification process included three major processes: (1) indicators were classified according to cut-offs defined; (2) each indicator was categorized into risk groups according to the determined cut-offs and scores assigned to each risk group; (3) the scores were summed per council across indicators, to obtain a combined measure that assigns the councils to the overall risk strata.

#### Classification definition of indicators

During the 1960s, various malariometric criteria were used to define geographical areas that should prepare for a pre-elimination stage, when community-based parasite prevalence (*Pf*PR) was consistently below 2–3% [29]. With time, this included indicators based on the prevalence of infections in fevers below 5% [30]. The current international guidelines for malaria elimination remain unspecific on the precise criteria for accelerating elimination efforts but define low transmission areas where community-based prevalence is between 1–10% and very low as below 1% [24]. WHO classifications of higher transmission settings include a moderate group (*Pf*PR 10–35%) and high (*Pf*PR > 35%) [24]. These continue to be arbitrary because the precise relationship between rates of infection, disease outcomes and optimized intervention remain poorly defined [31, 32].

For the stratification in mainland Tanzania, the classification has retained both very low (*Pf*PR_5–16_ < 1%) and high (adapted to be a *Pf*PR_5–16_ > 30%). Within this range, two additional groups were considered: low (*Pf*PR_5–16_ 1–5%) which provides a pre-very low classification to mitigate against the risks of misclassifying very low areas [33] and moderate prevalence (*Pf*PR_5–16_ 5–30%). There is far less historical evidence of appropriate criteria for the classification of fever infection prevalence and incidence, therefore, the prevalence in school children was used to guide the setting of appropriate cut-offs for categorizing these indicators (Table 2).

**Table 2:** Cut-offs used to categorize indicators into risk strata and scores assigned per epidemiological strata

Indicator^a^	Very low	Low	Moderate	High
School malaria parasitaemia survey				
Parasite prevalence				
Prevalence cut-off	< 1	1– < 5	5– < 30	≥ 30
Assigned score	1	2	3	4
Laboratory				
Fever test positivity rate				
Prevalence cut-off	< 5	5– < 15	15– < 30	≥ 30
Assigned score	0.5	1	1.5	2
Annual parasite incidence				
Prevalence cut-off	< 15	15– < 75	75– < 150	≥ 150
Assigned score	0.5	1	1.5	2
Outpatient Department				
Confirmed malaria incidence				
Prevalence cut-off	< 15	15– < 50	50– < 150	≥ 150
Assigned score	1	2	3	4
Antenatal clinic				
Test positivity rate				
Prevalence cut-off	< 1	1– < 3	3– < 10	≥ 10
Assigned score	1	2	3	4

^a^For information on the period of data used for each indicator, See Table 1

#### Risk categorization and assignment of risk scores per indicator

In a second step, all indicators for each council were categorized and assigned a score from 1 to 4 corresponding to four groups “very low (1)”, “low (2)”, “moderate (3)” and “high (4)” according to the cut-offs defined in Table 2. A pragmatic, conservative approach was taken that used the maximum of the annual mean values across the reporting years for each indicator per council, to assign councils to one of four strata. The aim was to increase the inclusion of councils that potentially are still at a higher risk to the high stratum, that will receive more control efforts, while avoiding assigning these high-risk councils into strata of reduced control efforts that might lead to rebound effects. For the laboratory indicators; API and RDT TPR, all available data in the observation period 2016–2017 were used. Since the overall laboratory reporting rates of health facilities in 2016 was only 49.6%, the assigned scores to these indicators were reduced in weight by an arbitrary factor of 0.5 to account for the low reporting rate.

#### Combination of indicators using scores

To obtain overall malaria risk by council, the sum of the assigned indicator scores was calculated. For each council, the resulting total score ranged from 4 (all indicators indicate “very low” malaria risk) to 16 (all indicators indicate “high” malaria risk). The scale from 4 to 16 was subdivided into four categories to form the epidemiological strata. Specifically, councils with an overall score ≤ 6 were allocated to the very low stratum, > 6 to ≤ 10 in the low stratum, > 10 to ≤ 14 in moderate stratum and > 14 in the high stratum (Table 2). In addition to these 4 epidemiological strata, urban councils were considered as a separate, non-epidemiological stratum with specific operational and intervention needs.

## Results

### Coverage and completeness

#### Survey data: SMPS

The SMPS was first conducted in 537 schools (49,169 school children) across 166 councils in 2014–2015 [11] and this was increased to cover 629 schools (66,823 school children) in 2017 to accommodate the expansion of administrative boundaries to 184 councils in 2016. During this period, the maximum annual mean prevalence in councils ranged from 0.0 to 76.4% (Table 3; see Additional file 2: Table S1). Of the 184 councils, 33 (18%) had malaria prevalence < 1.0%, whilst 80 (44%) councils had a high malaria prevalence ≥ 30.0%.

**Table 3:** Descriptive characteristics of the indicators used for malaria risk stratification

Parasite prevalence among school children [SMPS], 2015–2017	
No. councils	184
No. schools^a^	1166
No. children tested by Pf-Pan RDT	115,992
No. children with positive Pf-Pan RDT	21,382
Range of the maximum annual mean prevalence in councils [%]	0.0–76.4
Median prevalence [%]	20.9
Fever Test Positivity Rate [TPR] from Laboratory, 2016–2017	
No. councils	184
No. health facilities^a^	13,377
No. Pf-Pan RDT	22,848,520
No. positive Pf-Pan RDT	6,034,067
Range of the maximum annual mean prevalence in councils [%]	0.6–71.9
Median prevalence [%]	26.5
Annual Parasite Incidence [API] from Laboratory, 2016–2017	
No. councils	184
No. health facilities^a^	13,377
No. positive results by Pf-Pan RDT and microscopy	8,049,426
Annual population [projected 2017]	50,503,670
Range of the maximum annual mean incidence per 1000 population in councils	0.0–987.2
Median incidence per 1000 population	88.6
Confirmed Malaria Incidence from OPD, 2015–2017	
No. councils	184
No. health facilities^a^	21,644
No. confirmed cases by microscopy and Pf-Pan RDT in OPD	16,141,172
Annual population [projected 2017]	50,503,670
Range of the maximum of the annual mean incidence per 1000 population in councils	1.2–603.1
Median incidence per 1000 population	138.3
Test Positivity Rate from ANC, 2015–2017	
No. councils	184
No. health facilities offering ANC services^a^	18,513
No. ANC clinics that tested women	18,147
No. pregnant women tested by pf-pan RDT at first ANC visit	4,498,596
No. pregnant women with positive Pf-Pan RDT	321,836
Range of the maximum of the annual mean prevalence in councils [%]	0.1–29.2
Median prevalence [%]	8.8

*SMPS* School Malaria Parasitaemia Survey, *RDT* malaria rapid diagnostic test, *OPD* Out-patient Department, *ANC* Antenatal Care

^a^The number of facilities and schools are presented as the sum of all facilities/schools across the reporting years even if the same facility/school submitted data in the different years

#### Routine data: health facility data from HMIS/DHIS2

Table 3 summarizes the characteristics and coverage of the maximum annual mean values for the routine positivity rates and incidence indicators used for malaria stratification. In the period 2015–2017, a total of 212,311 facility monthly reports were received from 6437 health facilities offering ANC services resulting in an overall reporting rate of 92% across the councils. During this period, the maximum annual mean malaria prevalence in pregnant women ranged from 0.1 to 29.2% across the 184 councils (Table 3; see Additional file 2: Table S1).

Since the laboratory reporting tools were only introduced in health facilities in October 2015, data from laboratory registers in 2016 was received from health facilities in 178 councils, and by 2017, health facilities in all 184 councils submitted laboratory reports. A total of 107,486 monthly reports were received from 7188 health facilities resulting in an overall reporting rate of 62% during 2016–2017. Of the total malaria tests performed by both microscopy and pf-pan RDT, 8,049,426 were positive for malaria, showing a marked range in the maximum annual mean API from 0.0 to 987.2 per 1000 population per annum across the councils. During this period, the maximum annual mean fever RDT positivity rates ranged from 0.6 to 71.9% across the councils (Table 3; see Additional file 2: Table S1).

Monthly numbers of confirmed malaria cases in OPD were obtained from 7588 facilities across 184 councils in the period 2015–2017. Of the 273,168 expected monthly health facility OPD reports, 237,399 (87%) were received. During this observation period, there were a total of 16,141,172 cases of malaria reported from OPD resulting in the maximum annual mean malaria incidence ranging from 1.2 to 603.1 cases per 1000 population (Table 3, see Additional file 2: Table S1).

### Classification of indicators

**Fig. 1:**
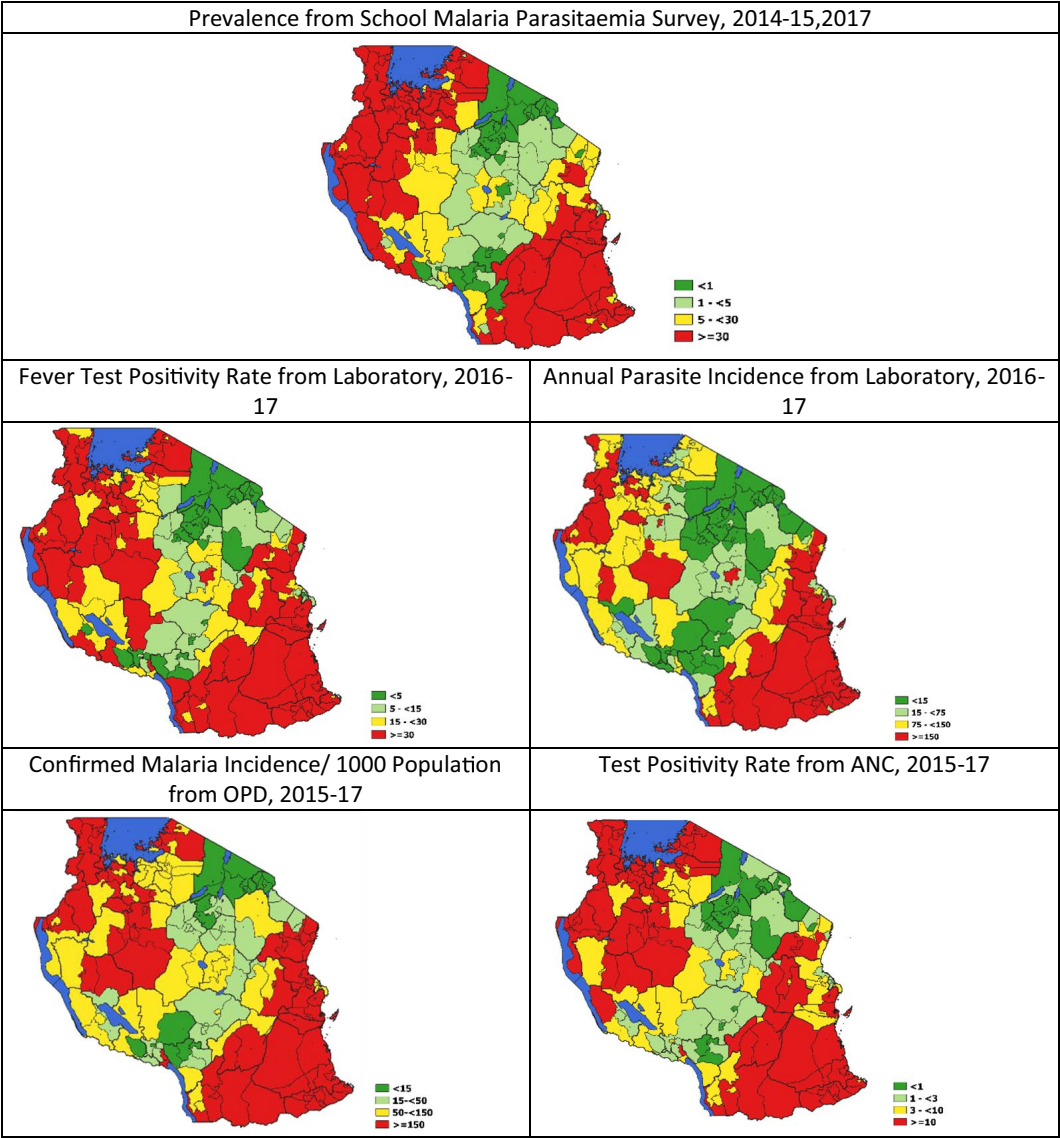
Spatial distribution by council of the maximum values of the mean annual malaria risk by type of indicator

Figure 1 shows the spatial distribution by council for the maximum of the average annual values for each of the malaria risk indicators for the period under review. Although variations exist between indicators in terms of the number of councils falling within each risk category, overall a similar pattern of heterogeneity was observed. The councils in the north-west and south east regions were consistently categorized into the moderate to high-risk groups while the councils in the central corridor running from northeast to south west were in the low and very low risk groups.

### Composite malaria risk stratification of councils

**Fig. 2:**
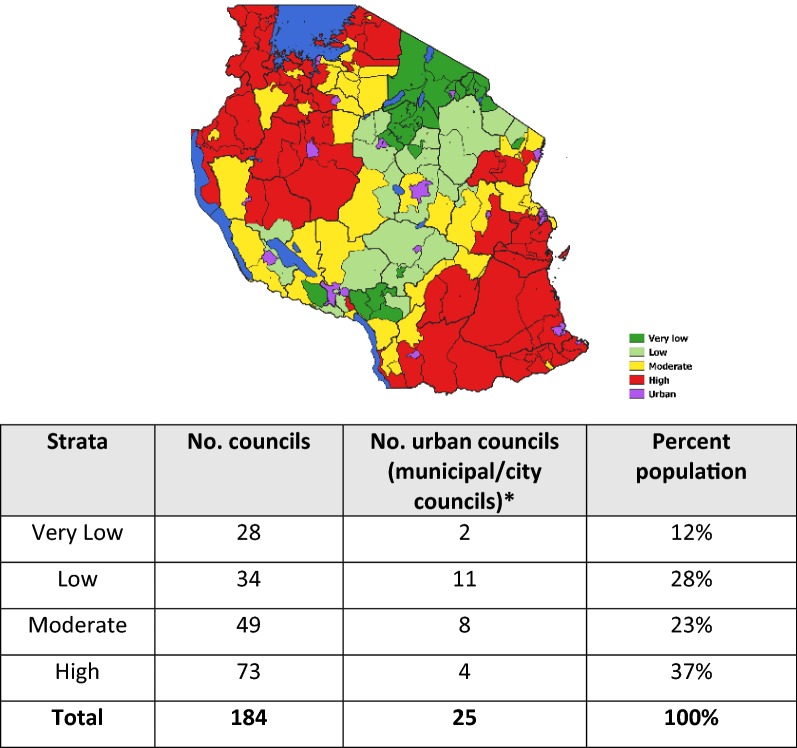
Overall distribution of councils by risk strata using the maximum of the mean annual values. *Urban councils in mainland Tanzania were considered as an additional non epidemiological stratum due to their specific operational and intervention needs

The final composite stratification map following the combination of the multiple malaria indicators is shown in Fig. 2. In the overall malaria stratification map of mainland Tanzania, 12% of the population resided in the 28 councils allocated to the very low strata, 28% of the population were in the 34 councils allocated to the low strata, 23% of the population resided in the 49 councils allocated in the moderate strata and 37% of the population resided in the 73 councils allocated to the high strata. Although all 25 urban councils were also assigned into one of the four strata, the urban councils were considered as an additional non-epidemiological stratum due to their specific operational and intervention needs (Fig. 2).

## Discussion

This paper presents a novel approach to stratify malaria at sub-national level in mainland Tanzania, using a combination of routine malaria indicators from health facilities and school surveys. The resulting map stratified the burden into four epidemiological risk strata; very low, low, moderate and high plus one non-epidemiological stratum for urban councils. This was used to guide the malaria control programme in revising its malaria strategic plan in an evidence-based manner and in developing targeted intervention packages per strata [18].

There are many indicators of malaria risk that can represent sub-national heterogeneity. The precision and bias of each indicator, associated costs for collection and the level and frequency available to measure variability across space and time can affect the suitability of indicators to measure transmission [34]. Several studies have attempted to compare measures from routine sources against community prevalence to highlight the representativeness of these indicators [25, 27, 35]. However, evidence to suggest which indicator is most suitable to measure transmission is limited and a further understanding of how these vary across different transmission settings would help identify which indicators are most sensitive to council-level transmission strata and how these change over time.

While there are several approaches to malaria risk stratification that have been developed, there is no one specific approach recommended by the WHO. A review that looked at malaria risk maps developed during pre-GTS, across 47 countries [36] found that most countries rely on either API or infection rates for describing the malaria risks although a range of other indicators have also been used such as qualitative descriptions and climatic suitability. The current methodology presents a pragmatic approach that levers data from routine reporting and national survey data. Not limiting the stratification to only one data source enhances the best use of all available data, and the credibility/robustness of the resulting stratification. Importantly, through a detailed interrogation of routine data, it is possible to make reasoned council indicators to align with other survey data sources for sub-national level stratification, harnessing data from those that seek care at facilities, attend ANC and schools nationwide.

Notably, two of these indicators, the malaria prevalence in pregnant women (from ANC clinics) and among school aged children (from school surveys), not available in many countries, contributed a uniquely rich source of information into the stratification for mainland Tanzania. The high attendance rates of pregnant women at ANC makes them an easily accessible surveillance population to track malaria transmission intensity and provides a simple routine real-time measure of malaria prevalence at higher spatial and temporal resolutions than national household surveys [37]. Prevalence from ANC clinics shows a correlation with community-based childhood infection prevalence [25, 27, 38] thereby serving as a good measure to reflect malaria trends in the community. Community-based malaria parasite prevalence has been a benchmark measure of malaria endemicity since the 1950s [29, 39] and used in Tanzania as a milestone for controlling progress since 2000s [7–9]. Since survey data obtained from national household surveys are not powered to provide information below regional levels, school-based surveys provide a rapid, cheaper alternative to household sample surveys [40, 41] and have been used in several countries during the 1960s [40, 42] to establish national malaria risk profiles. Tanzania’s investment into these two surveillance approaches was driven by the need for additional surveillance data as advocated by the GTS. While many countries do not conduct nationwide school surveys nor have a malaria surveillance established in ANC clinics, the basic principle of using other related data layers remains critical to developing a multilayered stratification. Countries might additionally include national household survey data, climatology or abiotic strata such as urban areas (as used in mainland Tanzania).

An important aspect to the methodology undertaken in mainland Tanzania is the simplicity of the design, without requiring complex modelling approaches often beyond the scope of those working within many national malaria programmes across Africa. The approach used was conservative, categorizing councils by their maximal risks over the past 2–3 years. Taking the maximum of multiple years’ data is valuable in ensuring that unstable councils prone to rebound of prevalence were not misclassified into the lower strata which improves the validity of the stratification and exposes more councils to aggressive control interventions. Statistical uncertainty is an important concept in risk mapping [43], but hard to interpret for many control programmes, and such a maximal-conservative use of data is one approach to a public health criterion avoiding “doing harm” [13].

The increasing availability of routine information from health facilities via DHIS2 offers an attractive scope for analyzing continuous epidemiological trends over time and monitoring service delivery at a frequency and level that is not possible through the national representative household surveys [44]. One of the most common criticisms for the use of HMIS data is the extent of the quality of the data reported through DHIS2, thereby leading to unreliable estimates of malaria risk [45]. However, as the reporting system in countries continues to improve, particularly following the launch of the High Burden to High Impact (HBHI) initiative that calls for improvements in HMIS system, the data will become increasingly more reliable. Recent evidence demonstrates the utility of these data, despite their inherent imperfections, for programme evaluations [46, 47].

There are obvious limitations to the use of routine data that could be improved with the use of new tools and better statistical handling of incomplete data. In the present approach, data from all health facilities were used, irrespective of their reporting rates. Additional file 1: Table S2 shows how the proportion of health facilities that can be included in the stratification varies depending on which threshold for reporting is applied. The influence on stratification when using only data from facilities with greater than 50% reporting rates is shown in Additional file 1: Figure S4. Applying a very strict criterion under which only data from facilities with complete reporting are included would mean that a small proportion of facilities could be included in the stratification. However, using a less stringent criterion, for example, including facilities with more than 50% reporting would increase the proportion of facilities that could be included in the stratification and was shown not to affect the overall strata allocation per council. Moreover, the arbitrary approach applied in setting appropriate cut-offs for classifying the routine indicators into the four risk groups questions the robustness of this approach. Defining accurate risk groups is crucial in ensuring that all councils are designated the correct strata.

Future work might include using all data with appropriate spatial interpolation techniques between missing months and missing reporting facilities [48] or consider the use of sentinel facility data with better reporting rates. Population distributions within councils are invariably uneven and assuming equivalent access to reporting facilities across a council could be improved with higher resolution population mapping, allowing for a more informed basis for facility-population catchments [49]. Furthermore, measures of incidence are influenced by a myriad of factors [50]. Novel techniques that adjust for treatment seeking behaviors have been developed and applied in malaria incidence estimation [51], however, these require complex models and simpler council-level adjustments are required for who seeks treatment from where [52]. Exploring the correlation matrices of the various routine indicators with each other and how they compare with community based prevalence is important in understanding the nature of the indicators in different transmission settings and defining robust and accurate thresholds for the classification.

Whilst the approach taken here has presumed equivalence between indicators, and a crude weighting applied to others (based on coverage), a more informed basis could be developed to maximize the relationships between indicators. In the absence of any formal guidelines to understand the representativeness, relatedness and appropriate cut-offs for individual strata, this is work planned over the next 3 years in mainland Tanzania. Meanwhile, the approach taken represents the most simplified means of handling multiple routine and survey composite data.

The stratification approach of mainland Tanzania served as a basis in guiding the malaria control programme in re-defining packages of interventions across the spectrum of malaria risk. No current guidelines exist as to which mix of interventions works best for which strata. In the absence of empirical evidence, using a data-driven approach guided by integration of impact modelling and expert recommendations, the country has developed the most suitable packages based on local context [12]. It is proposed to revise data inputs, approaches and strata every 3 years, as part of mid-term strategic reviews [18]. With increasing completeness of data, improved methodologies, and a changing impact of revised intervention, the process of stratification becomes dynamic.

Central health planning of malaria control in mainland Tanzania considers the council as the primary unit for resource allocation and policy. As the country moves towards implementing a targeted malaria control approach, a more granular stratification of malaria risk at sub-council level will become increasingly valuable in informing council health managers about their malaria situation. The wards will represent as important planning units especially when transmission intensity declines and stratification at this level will thereby support an evidence-based decentralized malaria control planning and implementation in mainland Tanzania.

## Conclusion

Mainland Tanzania has used a simple and novel methodological approach, combining multiple routine data sources with survey data for local, real-time monitoring of malaria risk at the council level. Whilst the data quality could still be further strengthened, it was sufficient to define and reflect the malaria risk heterogeneity across administrative boundaries. Using knowledge from multiple indicators of transmission increases confidence in stratification and allows for a baseline upon which the current national strategic plan might be judged.

## Supplementary information

**Additional file 1: Figure S1.** Administrative boundaries and distribution of urban and rural councils in mainland Tanzania. **Figure S2.** Locations of sampled schools for SMPS in 2015 & 2017 (N = 711). **Figure S3.** Location of operational health facilities by ownership in mainland Tanzania (N = 7620). **Table S2.** The cumulative proportion of health facilities submitting between 3 and 12 monthly facility reports from OPD, ANC and laboratory in 2015 and 2017 (N = Total number of facilities). **Figure S4.** Malaria risk stratification using health facilities with >50% reporting rates.

**Additional file 2: Table S1.** The maximum of the annual mean values per indicator and resulting overall risk strata assigned per council.

## Data Availability

Data from routine HMIS/DHIS2 as well as those from the SMPS are not publicly available and were obtained with request from the National Malaria Control Programme of mainland Tanzania. Restrictions apply to the availability of these data and permission can be obtained with reasonable request from the Ministry of Health, Community Development, Gender, Elderly and Children of mainland Tanzania.

## Abbreviations

ANC: Antenatal Care
API: Annual Parasite Incidence
DHIS2: District Health Information Software
GTS: Global Technical Strategy
HBHI: High Burden to High Impact
HMIS: Health Management Information System
M&E: Monitoring & Evaluation
MIS: Malaria Indicator Survey
MoHCDGEC: Ministry of Health, Community Development, Gender, Elderly and Children
RDT: Malaria Rapid Diagnostic Testing
NBS: National Bureau of Statistics
NMCP: National Malaria Control Programme
NMSP: National Malaria Strategic Plan
OPD: Out-Patient Department
*Pf*PR: *Plasmodium falciparum* Prevalence Rate
SMMSP: Supplementary Mid-term Malaria Strategic Plan
SMPS: School Malaria Parasitaemia Survey
TPR: Test Positivity Rate
UDSM: University of Dar es Salaam
WHO: World Health Organization
