# Supplementary material for: Sub-national stratification of malaria risk in mainland Tanzania: a simplified assembly of survey and routine data
Source: Malar J. 2020 May 8;19:177. doi: 10.1186/s12936-020-03250-4 (PMC7206674; doi:10.1186/s12936-020-03250-4)
Supplement: Supplementary file 1 — Additional file 1: Figure S1. Administrative boundaries and distribution of urban and rural councils in mainland Tanzania. Figure S2. Locations of sampled schools for SMPS in 2015 & 2017 (N = 711). Figure S3. Location of operational health facilities by ownership in mainland Tanzania (N = 7620). Table S2. The cumulative proportion of health facilities submitting between 3 and 12 monthly facility reports from OPD, ANC and laboratory in 2015 and 2017 (N = Total number of facilities). Figure S4. Malaria risk stratification using health facilities with >50% reporting rates. [file 12936_2020_3250_MOESM1_ESM.docx]

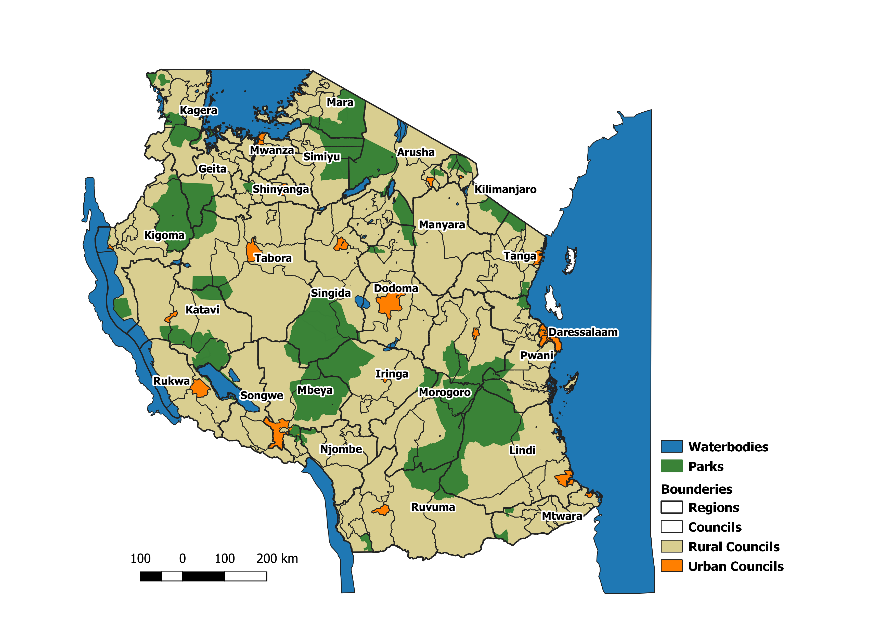


**Figure S1**: Administrative boundaries and distribution of urban and rural councils in mainland Tanzania. *Within the urban councils, town authorities were considered rural due to presence of high numbers of mixed and rural wards within the council thereby resulting in a total of 25 urban councils*


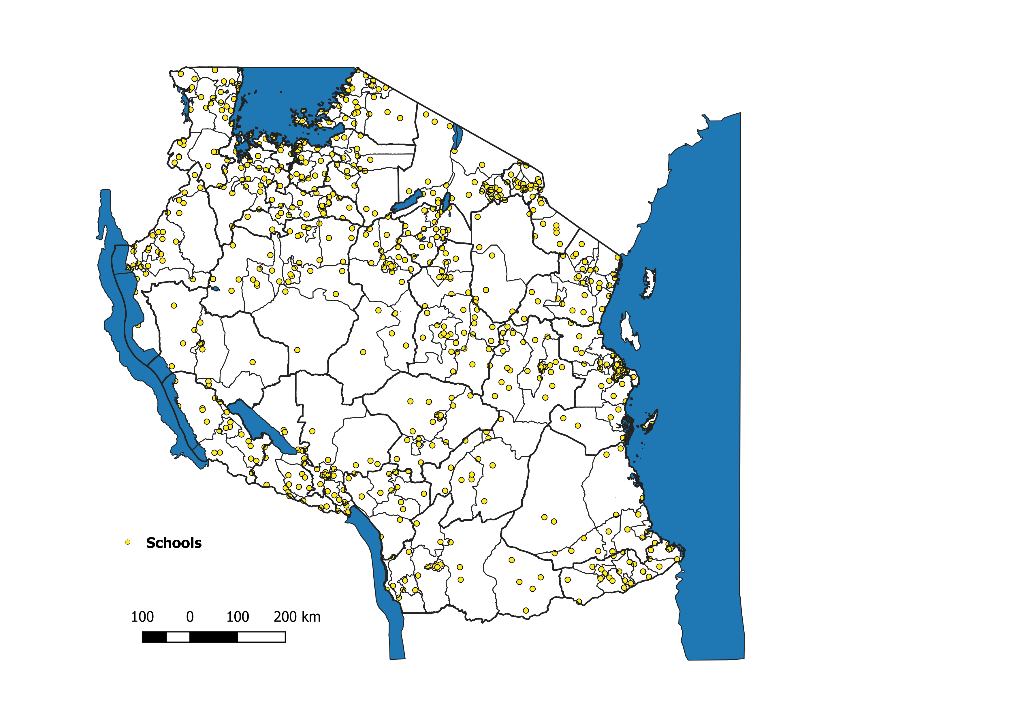


**Figure S2:** Locations of sampled schools for SMPS in 2015 & 2017 (N=711)


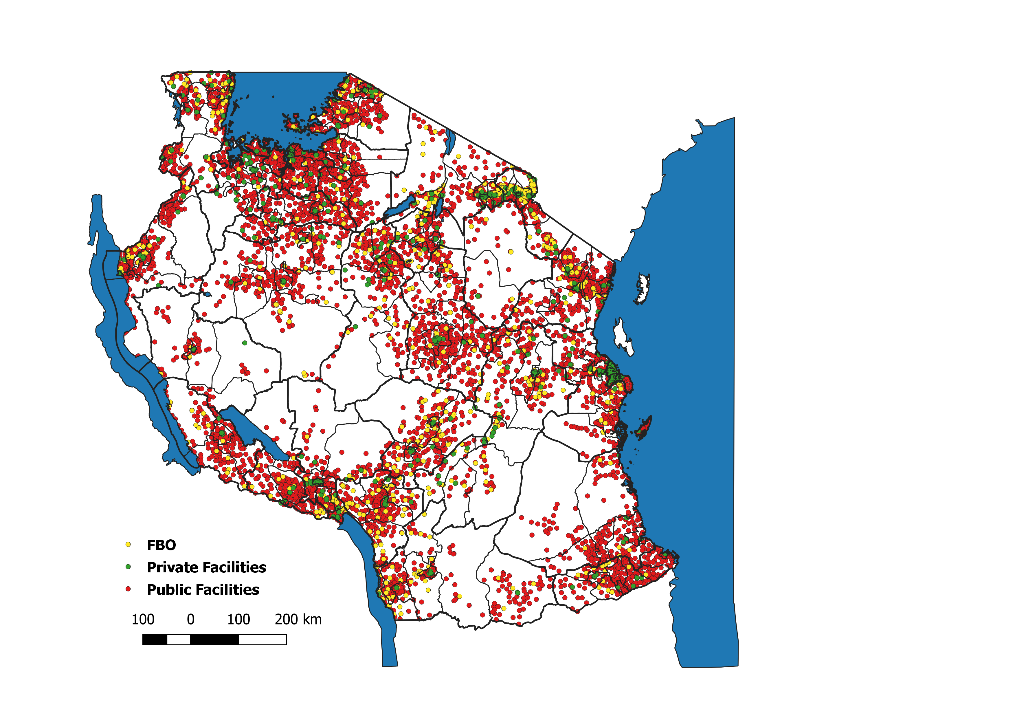


**Figure S3:** Location of operational health facilities by ownership in mainland Tanzania (N=7620) (Source: HFR Portal, www.moh.go.tz/hfrportal/)

**Table S1:** Excel file – The maximum of the annual mean values per indicator and resulting overall risk strata assigned per council

**Table S2:** The cumulative proportion of health facilities submitting between 3 – 12 monthly facility reports from OPD, ANC and laboratory in 2015 – 2017 (N = Total number of facilities)

| **# of monthly reports submitted** | **Laboratory** | | **OPD** | | | **ANC** | | |
| --- | --- | --- | --- | --- | --- | --- | --- | --- |
|  | **2016**  **(N=6297)** | **2017**  **(N=7078)** | **2015**  **(N=7004)** | **2016**  **(N=7215)** | **2017**  **(N=7425)** | **2015**  **(N=5981)** | **2016**  **(N=6170)** | **2017**  **(N=6362)** |
| **3** | 85.7% | 95.8% | 98.4% | 98.0% | 98.7% | 99.0% | 98.8% | 99.3% |
| **6** | 57.9% | 89.9% | 95.5% | 95.4% | 95.9% | 97.5% | 97.7% | 97.7% |
| **9** | 19.3% | 78.9% | 87.7% | 91.5% | 92.4% | 95.3% | 95.7% | 96.0% |
| **12** | 3.0% | 38.9% | 59.0% | 63.0% | 69.3% | 76.8% | 80.3% | 81.7% |

| 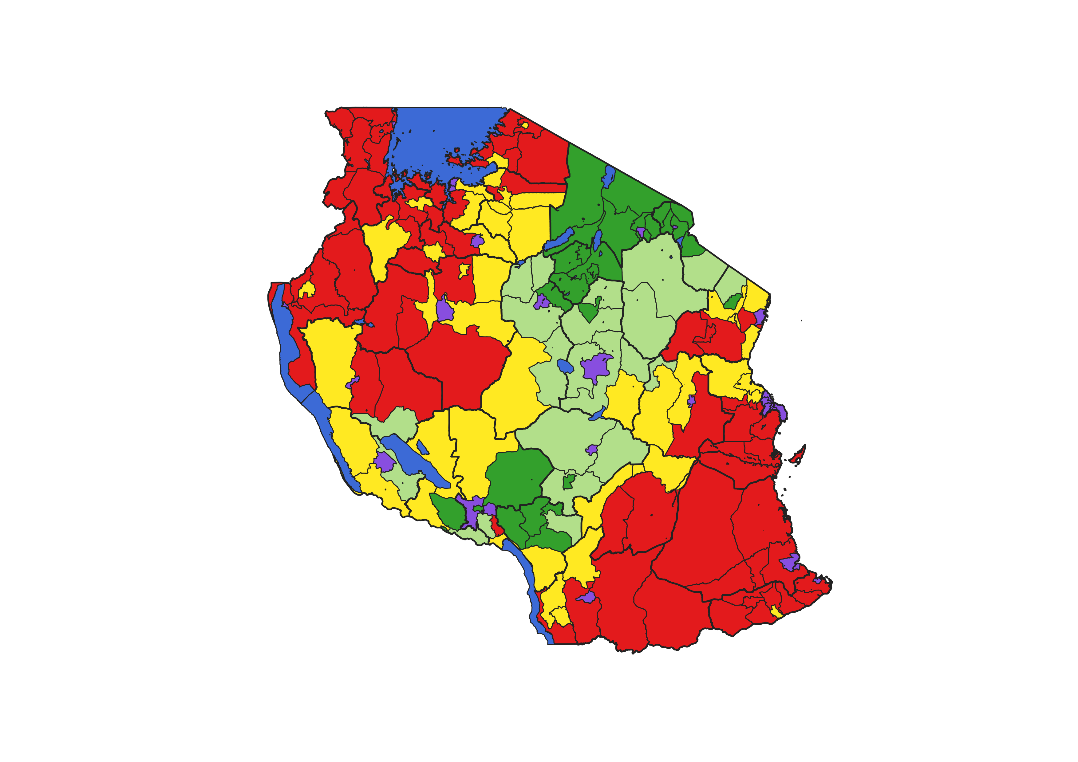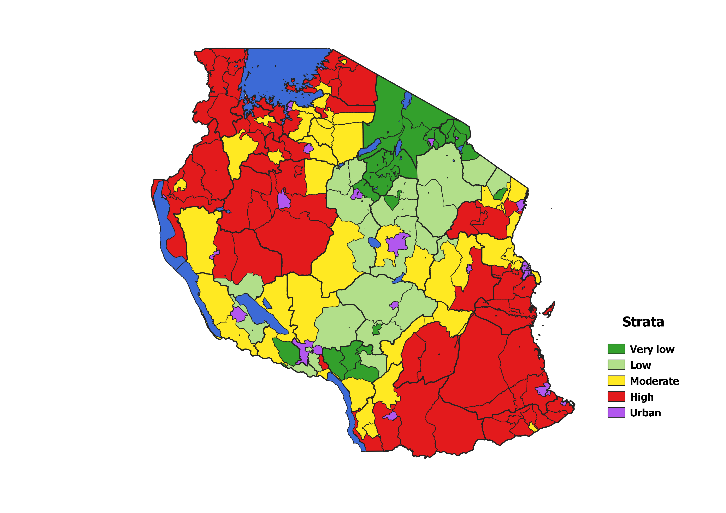 | \| **Strata** \| **No. councils** \| **No. urban councils (municipal/city councils)*** \| **Percent population** \| \| --- \| --- \| --- \| --- \| \| Very Low \| 31 \| 3 \| 36% \| \| Low \| 32 \| 10 \| 26% \| \| Moderate \| 50 \| 8 \| 24% \| \| High \| 71 \| 4 \| 14% \| \| **Total** \| **184** \| **25** \| **100%** \| |
| --- | --- | --- | --- | --- | --- | --- | --- | --- | --- | --- | --- | --- | --- | --- | --- | --- | --- | --- | --- | --- | --- | --- | --- | --- | --- |

**Figure S4**: Malaria risk stratification using health facilities with >50% reporting rates
